# Editorial: Understanding the Cytokine Release Syndrome: Toward Improving Cancer Immunotherapy 

**DOI:** 10.3389/fimmu.2021.666703

**Published:** 2021-03-19

**Authors:** Beatriz Martín-Antonio

**Affiliations:** Department of Experimental Hematology, Instituto de Investigación Sanitaria-Fundación Jiménez Diaz, Madrid, Spain

**Keywords:** cytokine release syndrome (CRS), immune effector cell associated neurotoxicity syndrome (ICANS), chimeric antigen receptor (CAR)-T cell, pro-inflammatory mediators, cytokines

## Introduction

Chimeric antigen receptor (CAR)-T cell immunotherapy, based on the infusion of genetically modified autologous T cells to recognize an antigen expressed on the tumor cell, has arisen in the last years as a new strategy to treat cancer patients. In B-cell hematological malignancies the administration of CAR-T cells has achieved outstanding responses leading to the FDA approval of two of these living drugs in 2017. Along with these remarkable results, toxicities known as cytokine release syndrome (CRS) and immune effector cell associated neurotoxicity syndrome (ICANS) develop in a subset of patients. These toxicities are mostly well-managed in a high proportion of patients. However, in some cases they can be fatal leading to death. Whereas development of CRS can occur in 100% of patients, neurotoxicity has been reported in more than 60% of patients (1). Of interest, analysis of data available from the FDA adverse event reporting system for patients treated with CART19 cells, reported that up to 7% of patients died due to non-relapse mortality within 30 days of initial CAR-T cell administration (2). Moreover, novel strategies that modify the CAR construct to increase the efficacy of CAR-T cells can involve an increased production of pro-inflammatory cytokines that might lead to higher incidence of CRS (3). These findings indicate the relevance to have a better understanding of these complications and to find novel targets to treat them and importantly, to find a balance to maintain CAR-T cell efficacy without increasing toxicity.

The development of CRS and ICANS starts when CAR-T cells attack tumor cells initiating a type of inflammatory cell death termed pyroptosis which leads to release of pro-inflammatory mediators, such as IL1β, that will activate macrophages leading to a massive release of cytokines (4). This massive release of cytokines by macrophages, which includes IL6, will lead to the development of CRS and ICANS. Currently, CRS is treated with tocilizumab, an anti-IL6R which neutralizes the IL6 released by macrophages. However, unlike IL6 which is one of the effector cytokines, targeting other initiator cytokines, such as IL1β, might provide with improved outcomes for these toxicities. Currently, mechanisms behind both complications are not totally well-understood. However, events leading to CRS are better characterized than those leading to ICANS. Of interest, ICANS often accompanies and correlates with CRS, being CRS and severity of CRS the most clearly defined risk factors for ICANS. However, ICANS can also occur independently from CRS and remains as an event with a high number of unknowns to be unraveled.

Currently, tocilizumab often resolves symptoms of CRS within hours and has become the standard of care and corticosteroids are being used to treat severe CRS if unresponsive to tocilizumab. However, patients refractory to tocilizumab and corticosteroids remain difficult to treat, which indicates the need to keep researching to find novel and specific targets to avoid CRS (5). On the other side, IL6 blockade is not effective for ICANS and in fact, it may worsen ICANS symptoms. Currently, ICANS is being treated with corticosteroids and different clinical trials are on-going to investigate other drugs, such as defibrotide, and blockade of IL1β and GM-CSF. Moreover, at pre-clinical level, novel strategies to avoid CRS and ICANS are being studied, such as a modification of the CAR construct to express a single chain variable fragment (scFv) directed against different cytokines (6).

At present, a high number of studies are being directed to understand to role of mediators leading to CRS and ICANS to unravel their pathophysiology. For both events, there is a variety of not only secreted mediators, such as IFNγ, IL1β, TNFα, IL6 and GM-CSF, but also different cells involved in the release of these mediators. Thus, whereas macrophages appear as the main cellular effectors inducing CRS, for ICANS, other cells involved include pericytes, myeloid cells and endothelial cells. Moreover, single-cell RNA sequencing data have also identified a rare cell population with monocyte-like transcriptional features associated with high-grade ICANS (7).

Another relevant factor that should be considered for CRS and ICANS is the impact that different organs can have in the severity of these toxicities. Thus, cytokines release during CRS cause extensive immunopathology to multiple vital organs, ultimately leading to their failure, which is clinically defined as multiple organ dysfunction (8). Thus, identifying whether the failure of any specific organ contributes more significantly to the severity of CRS than other organs can also help in more targeted and efficient treatment of CRS and ICANS. In this sense, CAR-T cell therapy and other inflammatory-related pathologies share T cell-driven CRS accompanied by multiple organ dysfunction, and studies in these other inflammatory-related pathologies may add interesting information for the treatment of CAR-T cell patients.

Intriguingly, not only pro-inflammatory mediators are involved in these events. Different molecules and cells well-known by their immunosuppressive role can be involved in the development of CRS and ICANS. Thus, IL10, an anti-inflammatory cytokine has been found to be involved in CRS (9) and in neurotoxicity (10) and their role in these events is starting to emerge. Moreover, myeloid derived suppressor cells, which are present in the tumor microenvironment, are well-known for their role inhibiting the cytotoxic activity of T cells. Whereas most studies suggest that the presence of these cells coincides with induced growth tumor and reduced activity of CD8+ T cells; it has been observed that myeloid derived suppressor cells stimulate IFNγ production by CD8+ T cells and promote CD69 and Fas upregulation in CD8+ T cells (11).

Last, an aspect that needs to be considered during CRS development is the impact that these pro-inflammatory mediators might have in the proliferation of residual tumor cells, since some of them are implicated in the pathogenesis of many cancers and it could be a double-edge sword in cancer treatment. In this regard, pro-inflammatory mediators such as ROS, FAS, CRP, TNFa, IL-1β and IL-6 are often implicated in different types of cancer. In particular, is well-known the role of IL-6 in ovarian cancer, breast cancer, and multiple myeloma due to its positive feedback loops that promote tumor growth (12–14).

To conclude, most studies on CAR-T cells have focused on the direct activity mediated by CAR-T cells against tumor cells, and the events activated by the vast range of secreted factors that occur upon tumor-CART cell interaction and their consequences remain poorly understood. With this Research Topic we aim to provide an overview of current knowledge of secreted and cellular mediators involved in the development of CRS and ICANS and to call readers’ attention towards the possible consequences that they might have.

## Author Contributions

The author confirms being the sole contributor of this work and has approved it for publication.

## Conflict of Interest

The author declares that the research was conducted in the absence of any commercial or financial relationships that could be construed as a potential conflict of interest.
